# Anti-Inflammatory Effect of Feiyangchangweiyan Capsule on Rat Pelvic Inflammatory Disease through JNK/NF-*κ*B Pathway

**DOI:** 10.1155/2018/8476147

**Published:** 2018-02-28

**Authors:** Yao Li, Yang Liu, Qian Yang, Zhihui Shi, Yanhua Xie, Siwang Wang

**Affiliations:** ^1^School of Pharmacy, Shaanxi University of Chinese Medicine, Xianyang 712000, China; ^2^College of Chemistry and Pharmacy, Northwest Agriculture and Forestry University, Yangling 712100, China; ^3^Department of Natural Medicine, School of Pharmacy, The Fourth Military Medical University, Xi'an 710032, China; ^4^Shaanxi Junbisha Pharmaceutical Limited Company, Shaanxi, China

## Abstract

**Objectives:**

In this study, we aimed to illustrate the preventive effect and possible mechanisms of Feiyangchangweiyan capsule (FYCWYC) on rat pelvic inflammatory disease (PID) model.

**Methods:**

To construct the rat PID model, upper genital tract was infected by multipathogen, and then drugs were orally administered for 8 days. The histological examination, immunohistochemical analysis, and ELISA were carried out. Furthermore, Western blotting was used to analyze the expression of Akt, MAPKs, NF-*κ*B p65, and I*κ*B-*α* in uterus.

**Results:**

As the results showed, infiltrations of neutrophils and lymphocytes in uterus were significantly suppressed, and IL-1*β*, IL-6, CXCL-1, and TNF-*α* were also reduced in a dose-dependent manner. We also found that FYCWYC inhibited apoptosis induced by infection. Furthermore, FYCWYC could block the infection-induced nuclear translocation of NF-*κ*B. We found that FYCWYC treatment only decreased the phosphorylation of JNK induced by infection and had no effects on Akt and P38. Additional, the effects of SP600125, an inhibitor of phospho-JNK, were similar to the results of FYCWYC.

**Conclusions:**

Taken together, our results demonstrated that FYCWYC had anti-inflammatory effect in pathogen-induced PID model, and the mechanism might be through inhibiting NF-*κ*B nuclear translocation which is mediated by JNK.

## 1. Introduction

Chronic pelvic inflammatory disease (PID), which is caused by ascending pathogenic microorganisms infection in the upper female genital tract, is a commonly observed distress in female genital system [1]. PID can lead to tubal factor infertility, ectopic pregnancy, chronic pelvic pain, and some others serious sequelae, and it has been considered a great threat to the women's life quality. Its symptoms include salpingitis, endometritis, and peritonitis [2]. The results from hematoxylin and eosin-stained experiment showed that there are many neutrophils and lymphocytes infiltrations in pathogenic sites, and this could be used as a criterion in diagnosing PID in clinic [3]. The common pathogens of PID include Gram-negative bacteria, mycoplasmas, trachomatis, Gram-positive bacteria, and* Neisseria gonorrhoeae* [4–6]. To treat PID, antibiotic regimens are often used in clinical practice, but antibiotics often induce antibiotic resistance [7]. Therefore, it is necessary to develop new complementary and alternative medicine for PID treatment.

Feiyangchangweiyan capsule (FYCWYC), a popular Chinese medicinal formula, is composed of* Euphorbia hirta *L.,* Polygonum chinense *L., and* Ilex rotunda *Thunb. In clinical settings, FYCWYC has been used in removing toxin, and treating bacteria induced bacillary dysentery, acute, and chronic gastroenteritis [8]. During patient follow-up, we found that many PIDs were alleviated, even cured. This information was very interesting, so we want to know the reasons.

Most of constituents of medicinal plants in FYCWYC have potential antimicrobial and anti-inflammatory activities.* Euphorbia hirta *L. exerts some pharmacological effects, including antipyretic, analgesic, and anti-inflammatory properties in rat and mice model [9]. The methanolic and ethanolic extracts of* Euphorbia hirta *L. also have antifungal and antibacterial activity [10]. Mah SH and his colleagues found that aqueous extract of* Polygonum chinense *L. had good effect in inhibiting inflammatory response [11]. Meanwhile,* Ilex rotunda *Thumb also had antibacterial and anti-inflammatory activities [12]. Bacterial infection and inflammatory are two main pathogenesis of PID; however, it is yet to be determined whether FYCWYC maintains strong antibacterial and anti-inflammatory activities and has some effects on PID. Pharmacological research of FYCWYC in lab may impart complete understanding of FYCWYC practice and more extensive application in China. In this study, we would observe preventive effect of FYCWYC on PID in rat model and further evaluates its pharmacological effect.

## 2. Materials and Methods

### 2.1. Materials

*Escherichia coli* (ATCC25922) and* Staphylococcus aureus* (ATCC25923) were purchased from American Type Culture Collection. Ultrapure water was obtained from a Milli-Q water purification system (Millipore, MA, USA). Detection kits for IL-1*β*, TNF-*α*, MCP-1, caspase-3, and IL-6 were obtained from Nanjing Jiancheng Bioengineering Institute (Nanjing, China). NF-*κ*B, I*κ*B, proliferating cell nuclear antigen (PCNA), Bcl-2, Bax, and *β*-actin antibodies were obtained from Cell Signaling Technologies, MA, USA. SP600125 (purity: 98%) was purchased from Sigma-Aldrich Company (St. Louis, Missouri, USA).

### 2.2. Preparation of FYCWYC

*Euphorbia hirta *L.,* Polygonum chinense *L., and* Ilex rotunda *Thunb were purchased from Zaolutang Co. (Xi'an, China) and identified botanically by Professor Haifeng Tang (Department of Natural Medicine, School of Pharmacy, Fourth Military Medical University). Three voucher specimens for these three herbs were deposited at the Department of Natural Medicine, School of Pharmacy, Fourth Military Medical University. All herbs (*Euphorbia hirta *L. :* Polygonum chinense *L. :* Ilex rotunda *Thunb = 4 : 2 : 1) were cut into pieces and soaked for 20 min in purified water (8 volumes) and then boiled and decocted for 20 min over a low heat. Repeat this process for two times; the decoction was homogenized and concentrated.

### 2.3. Rat PID Model Construction and Animal Treatment

Sprague Dawley (SD) rats, female, weighing 180–220 g, were purchased from the Experimental Animal Center of the Fourth Military Medical University. The experimental procedures were approved by the Ethics Committee for Animal Experimentation. All experiments were performed according to the National Institute of Health Guide for the Care and Use of Laboratory Animals (NIH Publications number 80-23, revised 1996) and the Guidelines for Animal Experimentation of the Fourth Military Medical University. 12 h dark/light cycles under a temperature of 22–24°C and a relative humidity of 60%–65% standard conditions were kept. SD rats were divided into six groups randomly (*n* = 8): control group (normal rats), PID group (rats infected by multipathogen), FYCWYC low-dose group (L, 0.6 g/kg), medial-dose group (M, 1.2 g/kg), high-dose group (H, 2.4 g/kg), and aspirin group (A, 2 mg/kg).

Rats were fed 7 days to adapt to the environment and then injected with progesterone (10 mg) subcutaneously. After these treatments, the PID model was constructed according to the methods reported previous [13]. Briefly, multipathogen solution which was composed of* U. urealyticum* (1 × 10^8^ ccu/ml) and* E. coli* (1 × 10^8^ ccu/ml) was prepared, and then absorbable gelatin sponge was immersed with 0.125 ml microbe-mixing solution. Multipathogen solution was inserted into rats' upper genital tract except control group, and then the rats were forced to stand upside down for 3 minutes. Four-time infections were conducted during 2 days. Microbe-free gelatin sponges with saline were inserted into control group rats. To detect the* U. urealyticum* and* E. coli* in vaginal swab samples, mycoplasma detection kit and TDR-300B automatic microbe analysis system (Tiandiren, Changsha, China) were used. FYCWYC and aspirin were given to rats orally for 8 days from the first infection. Eight days later, the rats were anesthetized with pentobarbital (30 mg/kg) subcutaneously. For the future experiments, uterus and fallopian tubes from experiment rats were collected and restored at −20°C. After these experiments finished, the rats were sacrificed by decapitation.

### 2.4. Histological Evaluation

For histological evaluation, the left uterus and fallopian tube of experiment rats were collected and cut into 2 *μ*m sections, fixed by buffered 10% formaldehyde for 24 h, dehydrated in alcohol, cleared in xylol, embedded in paraffin, and stained with hematoxylin-eosin at pH 3.5. Each slide was observed and evaluated randomly in 10 aleatory areas under a low-power microscopy field (×100) by two experienced pathologists.

### 2.5. Enzyme-Linked Immunosorbent Assay (ELISA)

Uterus and fallopian tubes in experiment rats were collected and weighed and then added to physiologic saline at the ratio of 1 : 5 (w/v) and then homogenized. The levels of C-reactive protein (CRP), interleukin-1*β* (IL-1*β*), interleukin-6 (IL-6), and tumor necrosis factor-a (TNF-a) in homogenate were measured by ELISA kits (Nanjing Jiancheng, Nanjing, China). The protein levels in tissue were measured by bicinchoninic acid (BCA) protein assay kit. The concentrations of the inflammatory cytokines were expressed as *μ*g/g protein of homogenate.

### 2.6. Western Blotting

The upper genital tract tissue samples were homogenized and lysed in ice-cold RIPA lysis buffer, mixed with SDS-PAGE sample buffer (2x), boiled for 10 min, and then resolved by 10% SDS-PAGE being transferred onto polyvinylidene difluoride (PVDF, Millipore, Billerica, MA, USA) membranes. Blots were blocked at 37°C for 60 min with 5% nonfat dry milk and then reacted with properly diluted monoclonal antibodies (1 : 1000) including NF-*κ*B p65, I*κ*B, Akt, P-Akt, P-P38, P38, P-JNK, JNK, PCNA, *β*-actin, Bax, and Bcl-2. The membranes were washed and incubated with horseradish peroxidase-conjugated goat anti-mouse IgG antibodies (1 : 1,000; Santa Cruz Biotechnology) at 37°C for 1 h, followed by enhanced chemiluminescence reaction (Pierce Biotechnology, USA).

### 2.7. Determination of Caspase-3 Activity

A fluorometric assay kit was used to measure the caspase-3 activity, and the protocol was according to the manufacturer. In brief, upper genital tract tissues of experiment rats were lysed in 50 *μ*l ice-cold lysis buffer for 30 min on ice and then centrifuged at 12000*g* at 4°C for 10 min. The protein levels in tissue were measured by BCA protein assay kit. The resulting fluorescence at 400 nm was measured by a microplate reader (Fluoroskan Ascent, Thermo Labsystems, Waltham, MA).

### 2.8. Statistical Analyses

The statistical data are presented as the means ± standard deviation (SD) of the indicated numbers of samples. The differences between two data sets were evaluated using Student's *t*-test by the SPSS 18.0 statistical software (SPSS, USA). One-way analysis of variance (ANOVA) was used to compare the difference between more than two data sets. *P* values of <0.05 were considered to indicate a statistically significant difference.

## 3. Results

### 3.1. Pathogens and Histological Examination

**Figure 1:**
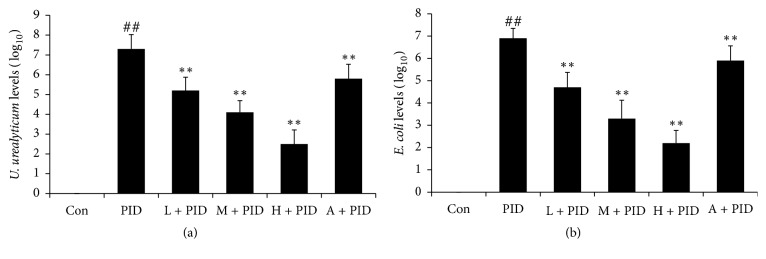
The antibacterial activity of FYCWYC was tested by pathogen examination. (a) The effects of FYCWYC on the* U. urealyticum* levels in the vaginal swab samples. (b) The effects of FYCWYC on the* E. coli* levels in the vaginal swab samples. Data were presented as the means ± standard error (*n* = 8). ^##^*P* < 0.01 versus control group, ^*∗∗*^*P* < 0.01 versus PID group.

To test the antibacterial activity of FYCWYC, pathogen examination was used. The results showed that vaginal swab samples from control rats showed negative results for* U. urealyticum* and* E. coli*, but vaginal swab samples from PID rats showed positive results for both, indicating successful infection in PID rats (Figure 1). The vaginal swab samples from FYCWYC treatment rats showed less* U. urealyticum* and* E. coli*, indicating that FYCWYC had antibacterial activity.

**Figure 2:**
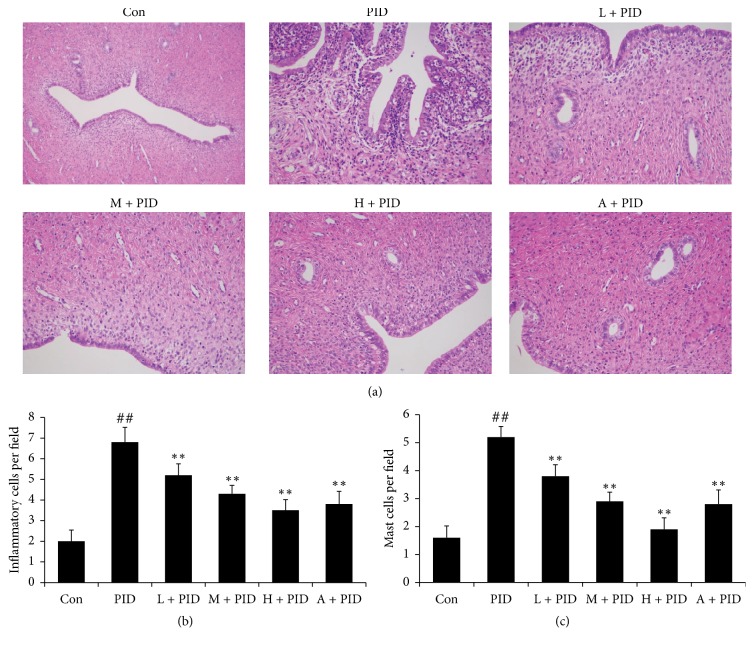
Effect of FYCWYC on infiltration of neutrophils and lymphocytes in uterus and fallopian tube after pathogenic infection. (a) Representative micrographs of uterus and fallopian. (b) Inflammatory cells numbers in the observed fields. (c) Mast cells numbers in the observed fields. Data were presented as the means ± standard error (*n* = 8). ^##^*P* < 0.01 versus control group, ^*∗∗*^*P* < 0.01 versus PID group.

Histological evaluation results were showed in Figure 2. In PID group rats, the uterus and fallopian tube were infiltrated by mass inflammatory cells, including neutrophils and lymphocytes. These results indicated that there was inflammation response in the upper genital tract. Compared with PID group, FYCWYC significantly decreased the inflammatory cells and mast cells infiltration in upper genital tract (Figure 2(a)). And the semiquantitative results also showed the significant differences between PID group and FYCWYC group on inflammatory cells (Figure 2(b)) and mast cells (Figure 2(c)) numbers.

### 3.2. FYCWYC Inhibited the Inflammatory Response in Rat PID Model

**Figure 3:**
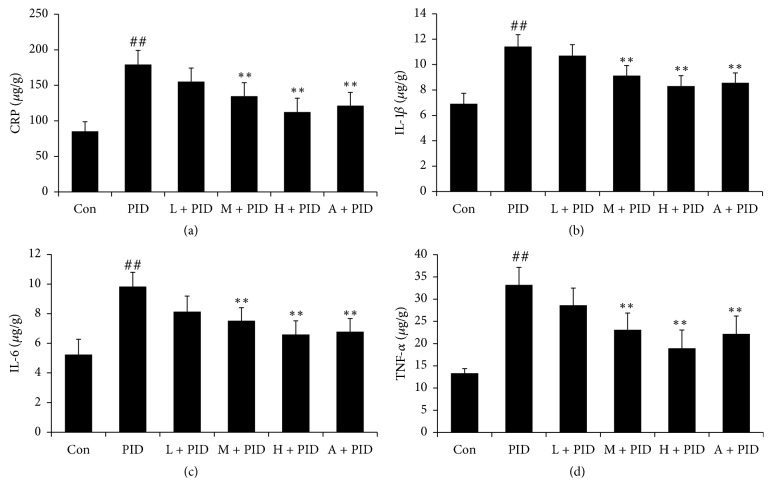
Effect of FYCWYC on the inflammatory response in PID model. (a) Effect of FYCWYC on CRP level in the upper genital tract after pathogenic infection. (b) Effect of FYCWYC on IL-1*β* level in the upper genital tract after pathogenic infection. (c) Effect of FYCWYC on IL-6 level in the upper genital tract after pathogenic infection. (d) Effect of FYCWYC on TNF-*α* level in the upper genital tract after pathogenic infection. Data were presented as the means ± standard error (*n* = 8). ^##^*P* < 0.01 versus control group, ^*∗∗*^*P* < 0.01 versus PID group.

To observe the inflammatory response, the IL-1*β*, IL-6, CRP-1, and TNF-*α* levels in PID rats were measured by ELISA kits. As the results showed in Figure 3, compared with control group, IL-1*β*, IL-6, CRP-1, and TNF-*α* levels were significantly increased in PID group, and FYCWYC significantly inhibited the production of these cytokines in rats. These results indicated that there was inflammatory response in PID rats and FYCWYC had anti-inflammatory effects.

### 3.3. FYCWYC Inhibited the Apoptosis in PID Model

**Figure 4:**
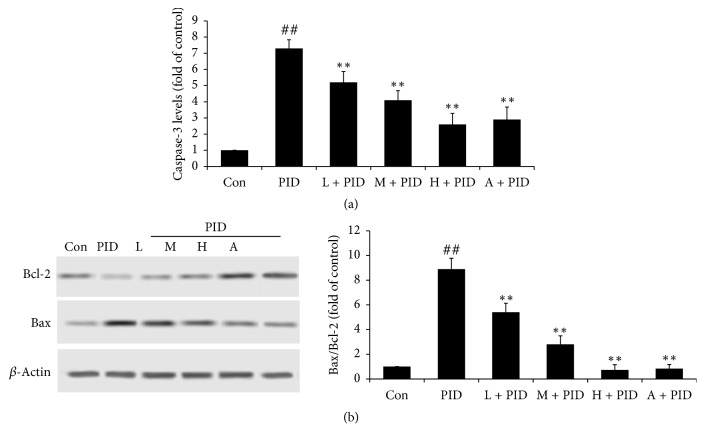
Effect of FYCWYC on the apoptosis in PID model. (a) Caspase-3 levels in the upper genital tract after pathogenic infection. (b) Bcl-2 and Bax protein expression levels in the upper genital tract after pathogenic infection. Data were presented as the means ± standard error (*n* = 8). ^##^*P* < 0.01 versus control group, ^*∗∗*^*P* < 0.01 versus PID group.

To determine the mechanism of FYCWYC, apoptosis in upper genital tract was measured. As the results showed in Figure 4, the caspase-3 levels and the Bax expression were significantly increased in PID rats and the Bcl-2 expression was significantly decreased compared with control group. Compared with PID group, the caspase-3 levels and the Bax expression were significantly decreased and the Bcl-2 expression was significantly increased. These results indicated that apoptosis was induced by bacterial infection and FYCWYC had some effects on apoptosis.

### 3.4. FYCWYC Inhibited the Expression of NF-*κ*B and I*κ*B-*α* in PID Model

**Figure 5:**
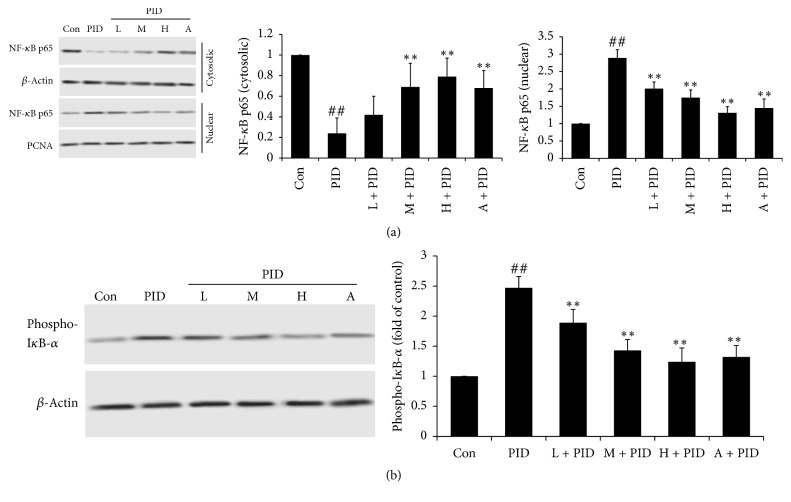
Effect of FYCWYC on the NF-*κ*B p65 and I*κ*B-*α* expression in PID model. (a) NF-*κ*B p65 levels in cytoplasm and nuclear were tested by Western blotting. (b) Phospho-I*κ*B-*α* in cytoplasm was tested by Western blotting. Data were presented as the means ± standard error. ^##^*P* < 0.01 versus control group, ^*∗∗*^*P* < 0.01 versus PID group.

To make clear the potential anti-inflammatory mechanism of FYCWYC, the expression levels of NF-*κ*B p65 and I*κ*B-*α* in nuclear and/or cytoplasm were determined by Western blotting. In PID group, NF-*κ*B p65 was translocated from cytoplasm to cell nucleus, and phospho-I*κ*B-*α* level in cytoplasm was more than that in control groups. In FYCWYC treatment groups, more NF-*κ*B p65 was distributed in cytoplasm, indicating that FYCWYC could inhibit the translation of NF-*κ*B p65 (Figure 5).

### 3.5. FYCWYC Inhibited the Phosphorylation of JNK Pathway

**Figure 6:**
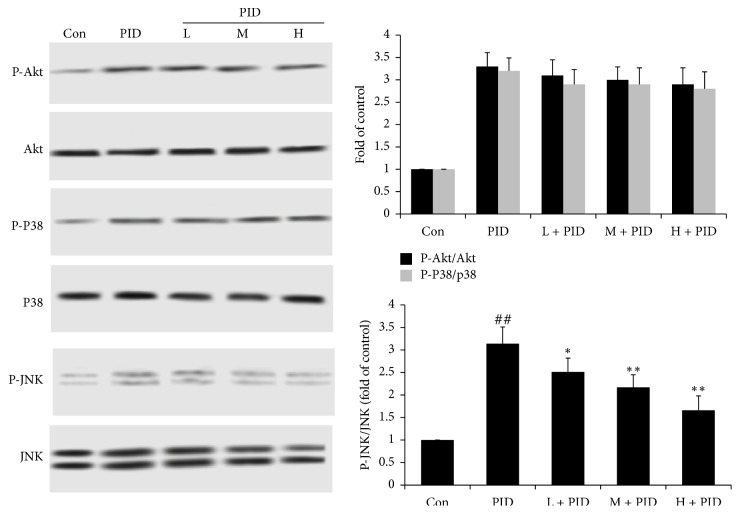
The effect of FYCWYC on the upstream of NF-kB pathway in PID model. The Akt phosphorylation, JNK phosphorylation, and p38 phosphorylation levels were tested by Western blotting. Data were presented as the means ± standard error. ^##^*P* < 0.01 versus control group, ^*∗*^*P* < 0.05, ^*∗∗*^*P* < 0.01 versus PID group.

To further study the upstream of Nf-*κ*B, the phosphorylation levels of Akt, p38 MAPK, and JNK were detected by Western blotting. As shown in Figure 6, we found that the phosphorylation levels of Akt, p38 MAPK, and JNK were increased in PID rats. Treatment with FYCWYC inhibited the increase of the JNK phosphorylation level. However, FYCWYC had no effect on the phosphorylation levels of Akt and p38. These results suggested that FYCWYC inhibited the infection-induced NF-*κ*B translocation via the inhibition of JNK.

### 3.6. FYCWYC Inhibited the Expression of NF-*κ*B through JNK

**Figure 7:**
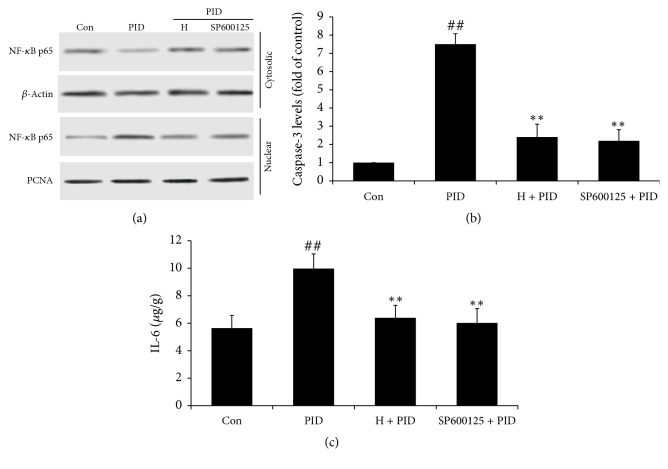
Inhibition of JNK phosphorylation by treatment by FYCWYC. (a) Rats were given an inhibitor of phospho-JNK, SP600125 (30 mg/kg) through intragastric administration. (a) NF-*κ*B p65 levels in cytoplasm and nuclear were tested by Western blotting. (b) Levels of caspase-3 were measured by caspase-3 kit. (c) IL-6 levels were measured by IL-6 kit. Data were presented as the means ± standard error. ^##^*P* < 0.01 versus control group, ^*∗∗*^*P* < 0.01 versus PID group.

Next, the JNK pathway was examined to investigate whether it was involved in the protective roles of FYCWYC in PID model. SP600125, a phospho-JNK inhibitor, was used to inhibit the phosphorylation levels of JNK (Figure 7(a)). The Western blotting results indicated that SP600125 inhibited IkB-a phosphorylation, reduced the translocation of NF-*κ*B from cytoplasm to nuclear, and thus decreased the NF-*κ*B activity. SP600125 also decreased the level of caspase-3 and IL-6 induced by infection (Figures 7(b) and 7(c)). These results indicated that the inhibition of phospho-JNK suppressed the activation of the NF-kB pathway and protected the cells from apoptosis and inflammation, which was similar to the role of FYCWYC.

## 4. Discussion

PID is a popular inflammation and infection in the women's upper genital tract, especially involving the uterus and fallopian tube. In some regions of US, PID is the most common gynecological reason in the hospital, about 18/10,000 recorded hospital discharges [14]. Among women with PID, about 40% develop into chronic pain, 20% become infertile, and 1% have an ectopic pregnancy [15]. In clinic, several pathogens which include* U. urealyticum*,* E. coli*, and some other Gram-negative and Gram-positive bacteria can lead to PID [16–19]. In this study,* U. urealyticum* and* E. coli* mixed solutions were used to promote inflammation in upper genital tract to simulate the PID model.

In clinic, broad spectrum antibiotics which are associated with high rates of short term improvement were always used to treat PID; however, there is an increased risk of tubal damage leading to chronic pelvic pain and infertility [20]. Nonsteroid anti-inflammatory drugs (NSAIDs) were also used in treating gynecological diseases, but NSAIDs play a limited role in the treatment once SPID forms, because of pelvic adhesion and fibrosis [21]. Long time and excessive use of NSAIDs also had obvious adverse effects, mainly involving ulcer, dyspepsia, and gastrointestinal bleeding, renal impairment, drug resistance, and weakened immunity [22, 23]. TCM had been used for thousands of years. Based on the thoughts of TCM, it is not only improving the main symptoms, but also addressing associated symptoms [24]. TCM has gained satisfactory effects in treating PID in the past years. In present study, we tried to clarify the protective effects of FYCWYC in treating PID and illuminate the possible mechanism.

In the present antibacterial examination, FYCWYC showed antibacterial activity in vivo. Many neutrophils and lymphocytes were found in the epithelium of upper genital tract after chronic pathogen infections, and FYCWYC significantly decreased the inflammatory cells infiltration in upper genital tract, indicating that FYCWYC inhibited tissue damage through inhibiting inflammation.

After pathogen infection in the body and recognition of immunogens by the TLRs, inflammatory response was initiated and propagated, and among this process, proinflammatory cytokines play essential roles [25–27]. As we known, IL-1*β* and IL-6 were two main proinflammatory cytokines. In upper genital tract of PID patients, elevated IL-1*β* could increase IL-6 level and in turn suppress the IL-1*β* synthesis in the second phase of the immune response [28, 29]. In upper genital tract, these proinflammatory cytokines can induce chemokines (CXCL-1, MCP-1) production which lead to hematopoietic immune cells recruitment and stimulate the proliferation and activation of leukocyte. Thus, neutrophils were activated and released inflammatory several cytokines and chemokines, which further intensify the inflammatory response [30]. In this study, we observed that the cytokines levels (IL-1*β*, IL-6, CRP-1, and TNF-*α*) in upper genital tract of PID rats were elevated, indicating inflammatory response was induced. Besides, treatment with FYCWYC significantly decreased these cytokines levels in a dose-dependent manner, suggesting a potent anti-inflammatory effect of FYCWYC.

Cell death can be found after necrosis and/or apoptosis. Necrosis and apoptosis were always accompanied by an aggressive inflammatory response and regulated many genetic and biochemical process [31]. As a response to infection, cell apoptosis has been observed in many pathogens infection [32, 33]. The caspase family of proteases plays important role in many aspects of apoptosis [34]. Caspase-3, a key enzyme in the downstream of apoptotic pathway, promoted apoptosis factors and then leads to apoptosis through caspase-3-mediated signaling pathways when it is active [35]. In this study, we found that caspase-3 levels were increased in PID group; however, in FYCWYC treatment groups, caspase-3 levels were significantly decreased. During infection in cells, Bcl-2 family proteins play important roles in the anti- and proapoptotic processes [36, 37]. In the present study, Bax and Bcl-2 were measured, and we found that Bax expression was increased and Bcl-2 expression was decreased. However, FYCWYC reversed those changes. These results suggested that apoptosis was induced in PID rats and inhibited by FYCWYC treatment.

NF-*κ*B, a crucial and ubiquitous transcriptional factor, plays important roles in regulating the transcription of several intercellular signaling pathways which was involved in inflammatory and immune responses [38]. Under normal condition, NF-*κ*B exists in the cytoplasm in an inactive complex with I*κ*B [39]. Upon infection, TLRs recognized pathogens and resulted in the phosphorylation and degradation of I*κ*B; then NF-*κ*B is free to translocate into the nucleus and promote the expression of many inflammatory mediators, such as IL-1*β* and IL-6 [40]. Those inflammatory mediators further activate NF-*κ*B and subsequently promote more proinflammatory mediators production [41]. Some pathogens that had been reported could activate NF-*κ*B, including* M. genitalium*,* M. fermentans*, and* M. pneumonia* [42–44]. In uterus and fallopian tube, NF-*κ*B p65 and I*κ*B are representative members of NF-*κ*B and I*κ*B family, respectively [45]. As showed in our results, multi-infection of pathogens caused the translation of NF-*κ*B p65 in uterus tissues. In the rat PID model, FYCWYC might exert its anti-inflammatory effects through blocking the NF-*κ*B pathway activation.

Many signaling pathways act as the upstream regulation protein of NF-*κ*B, like PI3K/Akt and MAPK signaling pathways. PI3K/AKT signaling pathway regulates a series of changes through lots of proteins including NF-*κ*B [46]. When PI3K/Akt was phosphorylated by some factors, IkB-a phosphorylation was induced and then leads to NF-kB activation [47]. It has been reported that MAPK, especially c-Jun NH(2)-terminal kinase (JNK), induces NF-kB activation at some conditions [48]. A variety of cellular stresses could induce and activate JNK and p38 MAPK, such as inflammatory cytokines [49]. JNK and p38 MAPK activation contributes to cell apoptosis and cell death [50]. Some researchers also found that virus infection could induce the activation of JNK and p38 MAPK and cause injury to patients [51]. However, there was little report about the relationship between MAPK and NF-kB in PID. In this study, we found that infection induced phosphorylation of AKT, JNK, and p38 MAPK, and FYCWYC only decreased JNK phosphorylation. An inhibitor of phospho-JNK, SP600125, was used to confirm its role in PID. We found that SP600125 inhibited the translation of NF-*κ*B p65 and cell inflammation and apoptosis induced by infection.

## 5. Conclusions

In this study, the preventive effect of FYCWYC against PID was found in a rat model, including reducing excessive production of inflammatory cytokines and inhibiting the infiltration of neutrophils and lymphocytes. Further, we also found that the potential mechanism of this effect might be associated with the suppression of JNK-NF-*κ*B pathway. The data from this study provided scientific foundation for the clinical uses of FYCWYC in alleviating PID infection and treating inflammatory disorders.
